# Multiple Oncogenic Pathway Signatures Show Coordinate Expression Patterns in Human Prostate Tumors

**DOI:** 10.1371/journal.pone.0001816

**Published:** 2008-03-19

**Authors:** Chad J. Creighton

**Affiliations:** Division of Biostatistics, Department of Medicine, Dan L. Duncan Cancer Center, Baylor College of Medicine, Houston, Texas, United States of America; Deutsches Krebsforschungszentrum, Germany

## Abstract

**Background:**

Gene transcription patterns associated with activation of oncogenes Myc, c-Src, beta-catenin, E2F3, H-Ras, HER2, EGFR, MEK, Raf, MAPK, Akt, and cyclin D1, as well as of the cell cycle and of androgen signaling have been generated in previous studies using experimental models. It was not clear whether genes in these “oncogenic signatures” would show coordinate expression patterns in human prostate tumors, particularly as most of the signatures were derived from cell types other than prostate.

**Principal Findings:**

The above oncogenic pathway signatures were examined in four different gene expression profile datasets of human prostate tumors (representing ∼250 patients in all), using both Q1-Q2 and one-sided Fisher's exact enrichment analysis methods. A significant fraction (∼5%) of genes up-regulated experimentally by Myc, c-Src, HER2, Akt, or androgen were co-expressed in human tumors with the oncogene or biomarker corresponding to the pathway signature. Genes down-regulated experimentally, however, did not show anticipated patterns of anti-enrichment in the human tumors.

**Conclusions:**

Significant subsets of the genes in these experimentally-derived oncogenic signatures are relevant to the study of human prostate cancer. Both molecular biologists and clinical researchers could focus attention on the relatively small number of genes identified here as having coordinate patterns that arise from both the experimental system and the human disease system.

## Introduction

Cancer is a disease characterized by DNA damage and widespread deregulation of cell signaling and gene transcription. Genes with roles in cancer can be broadly grouped into oncogenes, tumor supressors, and DNA damage-associated genes. Oncogenes promote cancer by over-expression or hyper-activation. The molecular pathways leading to oncogenesis and tumor progression are diverse [1]. Much progress has been made in the past 30 years in defining these pathways at the level of signal transduction involving protein-to-protein interaction. However, signal transduction leads through the chain of events to transcriptional regulation of a specific set of genes [1]. With the advent of global gene expression profiling technology within the last ten years, we can now more readily examine oncogenic pathways at the level of gene transcription. Through the use of experimental models such as cell cultures or transgenic mice, one may turn up the expression or activity of a specific gene and observe which genes are regulated as a result.

Here an oncogenic pathway signature will be defined to mean a set of genes that show a specific pattern of up- or down-regulation when a given pathway associated with oncogenesis is activated. Oncogenic signatures observed experimentally have potential use for inferring pathway deregulation in human tumors. In a seminal study by Lamb *et al*. [2], a set of genes induced by cyclin D1 in an *in vitro* model were found to be co-expressed as a group with cyclin D1 mRNA in multiple expression profile datasets of human tumors of various types. In a recent study by Bild *et al*. [3], gene signatures of Myc, Ras, E2F3, Src, and beta-catenin defined *in vitro* were used to predict Ras mutation status in human lung tumors and to predict the response of a panel of breast cancer cell lines to Src or Ras inhibitors. In another study by Creighton *et al*. [4], a signature of the MAP kinase pathway was defined from gene expression profiles of ErbB-2 (HER2), EGFR, Raf, and MEK in MCF-7 cells; this MAPK signature was found to share extensive similarities with signatures of ER-negative human breast cancer, which commonly has hyper-activated MAPK.

The oncogenic pathway signatures described above were generated from gene expression profiling of breast cell cultures. One question addressed here was which signatures could be considered relevant to human cancers of a different cell type from breast, in other words, whether genes associated with a given pathway in an experimental model show patterns of expression in human tumors that would be consistent with that pathway association. In the case of the Lamb cyclin D1 signature, human tumors of various cell types (including breast and prostate) that had high levels of cyclin D1 were found to express high levels of genes in the cyclin D1 signature [2]. This present study explored whether oncogenic signatures from other studies followed similar patterns in human prostate cancer. The focus of this study was in prostate cancer, as it is the most commonly occurring cancer in males in the United States, and as there were several profile datasets of human prostate cancer that were publically available.

Here, mRNA signatures of oncogenes Myc, c-Src, beta-catenin, E2F3, H-Ras, HER2, EGFR, MEK, Raf, MAPK, Akt, and cyclin D1, along with signatures of the cell cycle and of androgen signaling, were collected from eight previously published studies. As there have been multiple profiling studies of human prostate tumors [5]–[8], one can look for gene expression patterns that are common across independent datasets [9]. These oncogenic signatures were therefore examined in four different profile datasets of prostate tumors. As mentioned above, the aim of this study was to determine if these experimentally-defined oncogenic pathway signatures were relevant to human prostate cancer. The specific hypothesis tested was that human prostate tumors with (relatively) high mRNA levels of a given oncogene should also show high levels of the group of genes found over-expressed in the experimental setting when the same oncogene is turned up. One important outcome of this analysis was a catalog of the genes from a given experimentally-derived pathway signature that also had expression patterns considered relevant to the human tumors, which provides a resource for future functional studies.

## Results

### A collection of gene transcription signatures of oncogenic pathways from various studies

**Figure 1:**
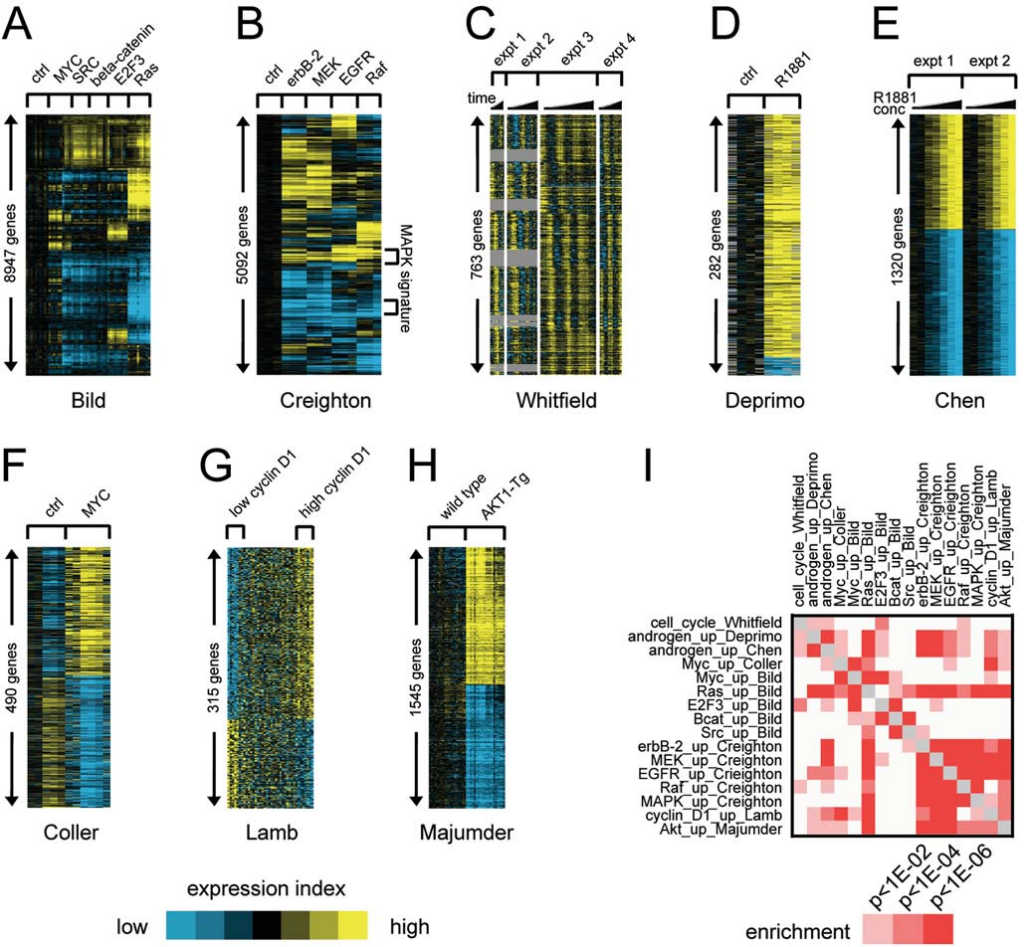
Gene expression patterns indicative of oncogenic pathway deregulation. RNA profiling datasets were collected from various published studies (described in Table 2) in which a particular pathway was activated in an experimental model. Expression patterns are represented as a color map. Each row represents a gene; each column represents a sample. The level of expression of each gene in each sample is represented using a yellow–blue color scale (yellow: high expression). Pathways represented are the following: (A) Myc, c-Src, beta-catenin, E2F3, and H-Ras, from the study by Bild *et al*. [3]; (B) erbB-2 (HER2), EGFR, MEK, Raf, and MAPK from Creighton *et al*. [4]; (C) the cell cycle from Whitfield *et al*. [10]; (D) androgen receptor (AR) signaling, from Deprimo *et al*. [11]; (E) AR signaling, from Chen *et al*. [12]; (F) Myc, from Coller *et al*. [13]; (G) cyclin D1, from Lamb *et al*. [2]; (H) Akt, from Majumder *et al*. [14]. (I) Graphical representation of the significance of gene overlap (by one-sided Fisher's exact test, using as the reference population the entire set of 14130 genes represented among any of the array platforms) between the various pathway signatures (focusing here on the sets of genes up-regulated in each signature).

Using gene expression profile datasets of cell culture and mouse models from previously published studies (listed in Table 1), gene signatures of 14 different oncogenic pathways were defined. These signatures are described in Table 2 and shown graphically in Figure 1. The pathways represented by these signatures were the following: (1) Myc, c-Src, beta-catenin, E2F3, and H-Ras, from the study by Bild *et al*. [3]; (2) erbB-2 (HER2), EGFR, MEK, Raf, and MAPK from Creighton *et al*. [4]; (3) the cell cycle from Whitfield *et al*. [10]; (4) androgen receptor (AR) signaling, from Deprimo *et al*. [11]; (5) AR signaling, from Chen *et al*. [12]; (6) Myc, from Coller *et al*. [13]; (7) cyclin D1, from Lamb *et al*. [2]; and (7) Akt, from Majumder *et al*. [14]. The unique named genes involved in each of these signatures are listed in Supplementary Data File S1.

**Table 1:** Public gene expression profile datasets used in this study.

Study	data source	array platform	# probes	# unique (human) genes
*Oncogenic signature datasets*				
Whitfield, et al. [10]	SMD	spotted cDNA	43896	14130
Deprimo, et al. [11]	SMD	spotted cDNA	33721	12934
Chen, et al. [12]	GEO (GSE846)	Affymetrix U133A	22283	12768
Coller, et al. [13]	Broad Institute	Affymetrix HUM6000-1,2,3,4	7252	3139
Bild, et al. [3]	GEO (GSE3151)	Affymetrix U133 Plus 2	54675	18134
Creighton, et al. [4]	GEO (GSE3542)	Affymetrix U133A	22283	12768
Lamb, et al. [2]	Broad Institute	Affymetrix HuGeneFL	7069	4990
Majumder, et al. [14]	Broad Institute	Affymetrix 430A	22691	11120
*Prostate tumor datasets*				
Glinsky et al. [5]	Oncomine	Affymetrix U133A	22283	12768
Yu et al. [6]	Oncomine	Affymetrix U95Av2	12625	8762
Lapointe et al. [7]	SMD	spotted cDNA	43844	14310
Singh et al. [8]	Broad Institute	Affymetrix U95Av2	12625	8762

SMD–Stanford Microarray Database (http://genome-www5.stanford.edu)

GEO–Gene Expression Omnibus (http://www.ncbi.nlm.nih.gov/geo/)

Broad Institute (http://www.broad.mit.edu/egi-bin/cancer/datasets.cgi)

Oncomine (www.oncomine.org)

**Table 2:** Oncogenic pathway gene signatures surveyed in prostate cancer (PCA).

Oncogenic pathway	Study	Model system	Number of unique genes (up)	Number of unique genes (down)	FDR
cell cycle	Whitfield, et al. [10]	synchronized HeLa cell cultures	763	NA	<1%
androgen	Deprimo, et al. [11]	treatment of LNCaP prostate cells with R1881	259	23	54%
	Chen, et al. [12]	treatment of LNCaP prostate cells with R1881	559	761	1%
Myc	Coller, et al. [13]	conditional Myc-estrogen receptor fusion protein in human primary fibroblast cells	252	238	9%
	Bild, et al. [3]	adenovirus infection of human primary mammary epithelial cells (HMECs)	993	1369	1%
Ras	Bild, et al. [3]	*ibid.*	1777	2286	<1%
E2F3	Bild, et al. [3]	*ibid.*	2029	1867	<1%
beta-catenin	Bild, et al. [3]	*ibid.*	976	1884	1%
Src	Bild, et al. [3]	*ibid.*	1566	1995	<1%
erbB-2	Creighton, et al. [4]	stable transfection of MCF-7 breast cancer cells	1315	1364	7%
MEK	Creighton, et al. [4]	*ibid.*	1238	1182	7%
EGFR	Creighton, et al. [4]	*ibid.*	734	940	11%
Raf	Creighton, et al. [4]	*ibid.*	618	988	11%
MAPK	Creighton, et al. [4]	*ibid.*	124	271	<1%
cyclin D1	Lamb, et al. [2]	adenovirus infection of MCF-7 breast cancer cells	206	109	22%
Akt	Majumder, et al. [14]	transgenic mouse prostate over-expressing human AKT1	770	775	1%

For each oncogenic signature, one set of genes were up-regulated and another set were down-regulated in response to activation of the associated pathway. Statistical criteria for defining each signature are given in the Methods section. Where selecting genes from profile data is a balance between false negatives and false positives, the selection cutoffs is this study leaned towards having fewer false negatives and more gene information. The *p*-value cutoffs used to define each signature were, in a sense, arbitrarily chosen, the idea being that a “sizable” number of genes (on the order of a few to several hundred) were desired to represent each pathway. Because of the wide spectrum of experimental systems, conditions, laboratories, and array platforms represented among all of the profile datasets, it was not possible to analyze all of the datasets in the same way and to use a single *p*-value cut off (e.g. *p*<0.01 for each comparison). There are many possible alternative methods and thresholds for defining the oncogenic signatures that might have been used and which could have been considered equally valid. The author believes that any reasonable analytic approach for defining signatures that yielded numbers of genes comparable to those in Table 2 would have resulted in the same overall patterns of enrichment being observed between the signatures and the human tumors.

Different oncogenic pathways may regulate common genes. The oncogenic signatures in this study were compared with each other to see which pairs of signatures shared significant gene overlap. These signature-to-signature associations are shown graphically in Figure 1I (focusing only on the set of genes up-regulated in each signature). A significant overlap of 40 genes between the set up-regulated by Myc in the Bild dataset (993 unique named genes) and the set up-regulated in the Coller dataset (252 genes) was observed (expected 18 genes, one-sided Fisher's exact *p*<1E-06, using as the reference population the entire set of 14130 genes represented among any of the array platforms), which shows good agreement between signatures of the same pathway generated independently by different labs using different experimental systems. Similarly, significant overlap was observed between the Deprimo and Chen sets of androgen-inducible genes (87 genes overlapping between 259 and 559 genes, respectively, one-sided Fisher's exact *p*<1E-57).

Furthermore, significant overlap was observed between signatures representing different pathways. The overlap between the Creighton erbB-2, EGFR, Raf, and MEK signatures had been noted previously [4], and was defined as a common MAPK signature. The overlap between the Bild Ras signature and the Creighton MAPK-associated signatures makes sense, as Ras is also a key intermediate of the MAPK pathway. In addition, the signature genes for cell cycle regulator E2F3 in the Bild data overlapped significantly with the set of genes correlated with cell cycle progression in the Whitfield data. Other signature-to-signature associations appear novel and intriguing, such as an association between the Akt pathway and the MAPK-associated pathways (Figure 1I). It is important to note that different signatures may share common genes, yet show widespread differences at the same time. For example, the Creighton MAPK-associated signatures shared a common MAPK signature, yet each of these signatures also had a distinct expression pattern from the other signatures (Figure 1B). The complete lists of corresponding genes overlapping among the various signatures are provided as Supplementary Data File S2.

### Genes up-regulated experimentally by Myc, c-Src, HER2, Akt, or androgen are co-expressed in human prostate tumors with Myc, c-Src, HER2, Akt, or PSA, respectively

The oncogenic pathway signatures of Table 2 and Figure 1 were derived from a number of experimental models, several of these signatures being derived from breast cell cultures in particular. An important question was whether these signatures would be relevant to the study of human prostate cancer, or would simply represent tissue-specific or model-specific effects on gene expression. Previously, cyclin D1-regulated genes from the Lamb profile dataset and Myc-regulated genes from the Coller dataset were each shown to be coordinately expressed with *CCND1* and *MYC* mRNA expression, respectively, in various tumor datasets [2], [15]. Here, the entire collection of oncogenic signatures from Table 2 was examined in each of four different expression profile datasets of primary human prostate cancers (PCA) from previous studies [5]–[8] (listed in Table 1), representing 253 individual prostate cases in all (Glinsky *et al*. dataset: 79 tumors, Yu: 60, Lapointe: 62, Singh: 52). Seven individual genes represented in the human datasets were of particular interest: *KLK3* (which encodes prostate-specific antigen, or PSA), *MYC*, *SRC* (which encodes c-Src), *ERBB2*, *EGFR*, *CCND1*, and *AKT1*. Each of these genes is an oncogene commonly over-expressed in cancers, with the exception of *KLK3*, a well-known gene target of androgen signaling. The hypothesis tested below with each of these seven genes was that prostate tumors with high expression (relative to other tumors) of either an oncogene or a biomarker of an oncogenic pathway would also show high expression of a significant number of genes up-regulated experimentally in the corresponding pathway signature. Additionally, these tumors with relatively high oncogene levels might show under-expression of a significant number of genes down-regulated in the pathway signature.

**Figure 2:**
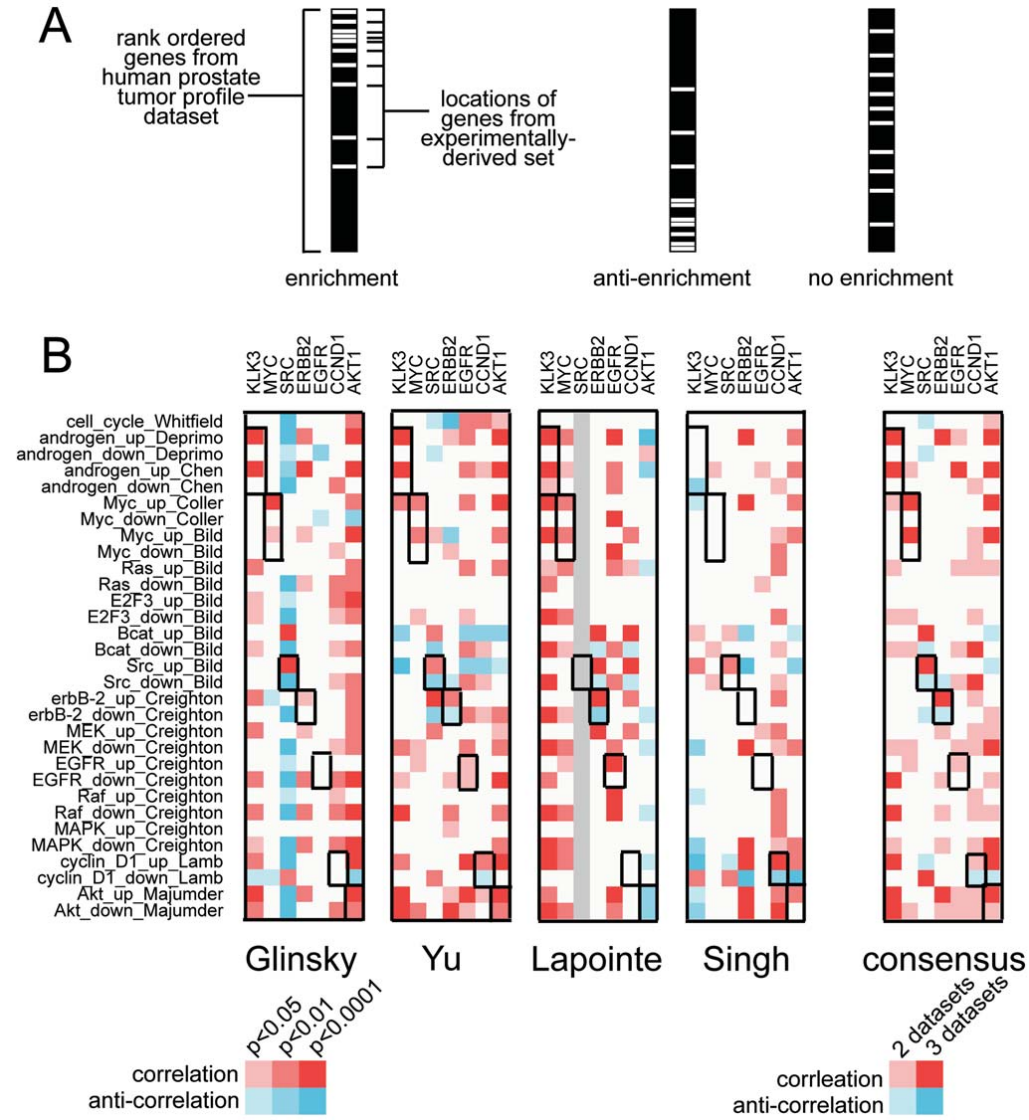
Patterns of enrichment involving selected oncogenes (*KLK3* i.e. PSA, *MYC*, *SRC*, *ERBB2*, *EGFR*, *CCND1* i.e. cyclin D1, and *AKT1*) and their corresponding pathway signature genes. (A) Schematic overview of Q1-Q2 enrichment analysis. Enrichment is defined as a significant number of genes in the experimental set being located at or near the top of a population of genes rank-ordered by a given metric applied to the human tumor dataset. (B) Results of enrichment analysis in four different gene expression profile datasets of human prostate tumors [5]–[8]. Patterns of consensus among the various datasets (i.e. patterns observed in at least 2 or 3 datasets) are also represented. For each oncogene-to-pathway association, a red square indicates that genes in the pathway were enriched within the top genes most correlated with the oncogene. Anti-enrichment (blue square) indicates that genes in the pathway overlapped with the genes most anti-correlated with the oncogene. Associations between a given gene and its corresponding oncogenic pathway signature are highlighted with black outline.

For each of the seven oncogenes mentioned above, the genes most correlated (i.e. similar) in expression to the given oncogene were determined in each of the four prostate datasets. Gene correlates of the oncogene were tested for enrichment of each of the 16 oncogenic pathway signature gene sets of Table 2. As each pathway signature had a set of genes up-regulated and another set down-regulated in response to pathway activation, the “up” genes were evaluated for enrichment separately from the “down” genes. The test of enrichment used here was the rigorous, rank-based “Q1-Q2” statistic of Tian *et al*. [16] (schematic in Figure 2A), which demonstrated significance of enrichment over what may be expected using a randomly-generated set of pathway signature genes (the “Q1” hypothesis) or using a random ordering of reference oncogene values in the human tumor dataset (the “Q2” hypothesis). Besides the seven oncogenes of interest, other genes, such as genes encoding MAPK or beta-catenin, were represented by pathway signatures, though many of these other genes are typically mutated or hyper-activated in cancer, rather than over-expressed.

Results of the enrichment analysis are shown in Figure 2B. A major advantage in evaluating four independent prostate tumor datasets is that one may identify consistent patterns appearing in multiple datasets. Numerous patterns of enrichment were found in three of the four datasets examined (though none were found in all four). The major finding of the enrichment analysis was that genes up-regulated experimentally by Myc, c-Src, HER2 (erbB-2), Akt, or androgen were consistently co-expressed in human PCA with Myc, c-Src, HER2, Akt, or PSA, respectively. Associations of the cyclin D1 and EGFR pathway signatures with expression of *CCND1* and *EGFR*, respectively, were also observed in two of the four tumor datasets. Many of the gene sets from different oncogenic signature pathways shared significant overlap (Figure 1I), yet, interestingly, each pathway set associated uniquely with its corresponding oncogene; for example, the EGFR and erbB-2 signatures had many genes in common (Figure 1B), yet the EGFR signature genes did not associate with mRNA patterns of erbB-2 in the human data, and vice versa (Figure 2B). Another notable finding is that genes down-regulated in a given oncogenic signature did not show anticipated patterns of anti-enrichment.

The Q1Q2 enrichment analysis of Figure 2B was a ranked-based approach, where genes that were even weakly correlated with the reference gene of interest could partially contribute to a significant association (see ref [16] for details). Therefore, an alternative enrichment method, the classical one-sided Fisher's exact test, was also considered. For each of the androgen, Myc, Src, HER2, EGFR, cyclin D1, and Akt pathways, the top genes overlapping between the oncogenic signature and the correlates of the corresponding oncogene or biomarker in the human tumors were tabulated. The numerical results of the tabulation are given in Table 3, and the lists of genes overlapping between the experimental and human datasets for each pathway signature are given in Supplementary Data File S3. For Table 3 and Supplementary Data File S3, positive correlation between a gene and the given oncogene within the human tumors was defined as showing statistical significance (*p*<0.05, Pearson's correlation, two-sided) in at least three out of the four prostate profile datasets. Genes listed in Supplementary Data File S3 were therefore both in the given pathway signature (as defined in Table 2) and individually correlated with the corresponding oncogene or biomarker in the human tumors.

**Table 3:** Number of genes overlapping between oncogenic signatures and human tumor correlates.

Oncogenic signature	Signature genes*	Prostate tumor genes**	Expected overlap	Actual overlap#	P-value##
androgen_up_Chen	428	176	9	29	1.2E-08
Myc_up_Bild	475	150	8	33	7.9E-12
Src_up_Bild	767	116	11	23	0.0003
erbB-2_up_Creighton	1011	307	37	91	1.3E-17
EGFR_up_Creighton	560	28	2	1	0.85
cyclin_D1_up_Lamb	203	77	3	8	0.02
Akt_up_Majumder	512	262	20	47	3.6E-08

* Number of signature genes represented in at least three out of four prostate profile datasets.

** Number of genes positively correlated with corresponding oncogene/biomarker (see Figure 2A) in human prostate tumors (Criterion: p<0.05 in at least three out of four profile datasets).

# Lists of overlapping genes included in Supplementary Data File S3.

## By one-sided Fisher's exact test, using, as the reference population, the set of unique genes common to both the oncogene signature array platform and any three of the prostate tumor array platforms (Chen, Creighton: 8500; Bild: 8402; Lamb: 4731; Majumder: 6560).

Except for the EGFR signature, there was non-random overlap between the set of genes in the experimentally-defined signature and the set of genes correlated in the human tumors with the reference oncogene or biomarker corresponding to the signature. For example, of the 559 genes in the Chen androgen signature (448 of which were represented in at least three prostate tumor datasets), 29 were correlated (*p*<0.05) with *KLK3* in at least three out of the four prostate tumor datasets; by chance, nine were expected, and so the amount of actual overlap was quite significant (*p* = 1.2E-08). The overlap was not significant for the EGFR signature and was marginally significant (*p* = 0.02) for the cyclin D1 signature. The results of the Fisher's exact enrichment analysis (Table 3) appear consistent with the results of the Q1Q2 enrichment anlysis (Figure 2B), where the *EGFR* and *CCND1* analyses were significant in only two of the four datasets; relatively few genes (28) were individually correlated with *EGFR* mRNA in at least three human tumor datasets, and genes that were correlated in only two datasets would not have been included in the Fisher's exact analysis.

## Discussion

In this study, several gene signatures of oncogenic pathways defined experimentally were found to be coordinately expressed with the single oncogene or biomarker corresponding to the pathway in human prostate tumors. These results demonstrate these signatures to be relevant to the study of human prostate cancer. These findings apply mainly for the sets of genes up-regulated in the signatures, as the down-regulated genes by and large did not show expected correlation patterns (Figure 2B). The gene-to-signature correlations of interest were observed in three of the four prostate tumor profiles datasets, but none were found in all four. The fact that no consistent patterns were found in all four datasets is curious but could be explained by a number of reasons, which could range from the technical (e.g. a “bad” gene probe or chip artifact in one dataset) to the biological (e.g. different patient cohorts being represented in different datasets). It should be emphasized that any association made between a gene and its corresponding signature for any one dataset was quite robust, the significance values testing against 1000 randomly-generated gene signature and 1000 permutations of the reference oncogene values (see Methods). This study does underscore the value of the meta-analysis approach, as patterns that may be missed in one profile dataset could be repeatedly found in other datasets.

In general, experimental data might be expected to show changes in gene expression that are merely artifacts of the model system, while human tumor data alone can show patterns of correlation but do not readily demonstrate cause-and-effect in the way experimental models can. For a given pathway, the intersection of the set of genes up-regulated by experimental activation of the pathway with the set of genes showing anticipated patterns of expression in human tumor tissue specimens would be a set of genes of potential interest to researchers. For the Myc, c-Src, HER2, EGFR, cyclin D1, Akt, and androgen pathways, this study provides the set of genes relevant to each pathway both experimentally and pathologically (Table 3 and Supplementary Data File S3). Molecular biologists who study any one of these pathways may select candidate gene targets of these pathways uncovered by this study and validate the expression patterns both in experimental models (models using prostate cells in particular) and in human prostate tumor specimens.

It is understood that a particular pathway may be deregulated through a number of different genes [1], and so a measure of the “end point” of a signaling pathway at the transcription level may prove to be a better indicator of pathway deregulation over any single gene. The intersection of human and experimental profile data provides sets of genes that could possibly be used in a clinical diagnostic assay to infer which pathways are deregulated in patient tumors. RT-PCR assays using RNA from paraffin-embedded tissues have been developed [17], and so it would be technically feasible to develop an RT-PCR-based, multi-gene assay for the status of various pathways in a given tumor, using genes selected from the results of this study.

The analysis presented here implicates over-expression of Myc, Akt, c-Src, or erbB-2/HER2 with activation of their associated pathways in at least a subset of prostate tumors. The roles of Myc and Akt in prostate cancer initiation and progression have received much study [18], [19], [14]. The c-Src oncogene has apparently not received much attention in prostate cancer and is better known in other cancers such as colon [20]. However, a truncated version of the c-Kit prostate was recently found in primary prostate tumors, which was correlated with activation of the Src pathway [21]. The role of erbB-2 is prostate cancer has been controversial, and there have been conflicting results over whether the gene is amplified or over-expressed [19]. This present study indicates that there is a sub-population of prostate cancer patients that express erbB-2 and have its pathway activated; such patients could possibly benefit from current anti-erbB2 therapies, such as Herceptin, currently in use in breast cancer.

Other recent molecular profiling studies (in addition to those referenced above) have demonstrated that gene expression patterns derived from experimental models are also observable in human tumors. For instance, transgenic mice over-expressing *Myc* shared many of the expression patterns observed in human prostate tumors having high *MYC* expression [22]; a mouse model of Kras2-mediated lung cancer shared expression patterns with human lung tumors harboring k-ras mutation [23]; and an SV40 T/t-antigen cancer gene signature activated in transgenic mouse tumors with aberrant p53, Rb, or BRCA1 expression was associated with poor prognosis in human breast, prostate, and lung carcinomas [24]. In this context, the present study represents a validation of the idea that gene expression patterns derived experimentally can be relevant to the study of human cancer at the transcriptomic level.

## Materials and Methods

### Gene expression profile datasets

The gene expression profile datasets described here were publicly available [2]–[8], [10]–[14] (Table 1). Gene expression values were log-transformed. For the Chen androgen dataset [12], expression values within the AR+ group of samples were transformed to standard deviations from the mean; values within the vector group of samples were separately transformed. Treatment of the Coller *et al*. dataset [13] is described in ref [15]. Human prostate tumor datasets were transformed to standard deviations from the median. For the Lapointe *et al*. cDNA microarray dataset [7], gene probes with missing values in at least half of the tumor specimens were removed from consideration. From the 66 PCA profiles described in the study by Yu *et al*. [6], 60 were available for this study (collection for this dataset was facilitated by A.M. Chinnaiyan and the Oncomine team); the “adjacent tissue” (AT) profiles from the Yu study were not considered here. Expression values were visualized as heat maps using the Cluster [25] and Java TreeView software [26].

### Definition of oncogenic pathway signatures

Two-sample *t*-tests determined significant differences in gene expression between groups of samples. Criteria for selection of genes in each oncogenic pathway signature was as follows: (1) Myc signature from Bild dataset, *p*<0.001, comparing the Myc group with GFP control; Bild Ras signature, *p*<0.00001; Bild E2F3 signature, *p*<0.001; Bild beta-catenin signature, *p*<0.001; Bild Src signature, *p*<0.0001; erbB-2 (HER2) signature from Creighton dataset, *p*<0.01, comparing HER2 group with MCF7/lt-E2 control; Creighton EGFR signature, *p*<0.01; Creighton MEK signature, *p*<0.01; Creighton Raf signature, *p*<0.01; Creighton MAPK signature, described in ref [4]; Whitfield cell cycle signature, described in ref [10]; androgen receptor (AR) signaling from Deprimo dataset, *p*<0.01, comparing R1881-treated LNCaP samples with EtOH controls, and a minimum average red/green ratio of 1.8 in R1881 samples; AR signaling from Chen dataset, *p*<0.001 for Pearson's correlation with log of R1881 concentration; Coller Myc signature, described in ref [15]; Lamb cyclin D1 signature, *p*<0.01 for Pearson's correlation with *CCND1* mRNA expression across all samples; Majumder Akt signature, *p*<0.001, comparing AKT-Tg mouse prostate with wild-type (placebo-treated groups). False Discovery Rate (FDR) was computed for each oncogenic signature, using the method of [27]; the number of probes on the array was multiplied by the nominal *p*-value and divided by the number of probes in the oncogenic signature (FDR values listed in Table 2).

### Enrichment analyses

In order to determine whether a specified group of experimentally-derived genes (e.g. genes induced in the Myc signature) had a coordinated association with a molecular phenotype of interest in human tumors (e.g. prostate tumors with high *MYC* expression relative to the other tumors), Q1-Q2 analysis was carried out essentially as described in [16]. Briefly, the common population of genes represented in the given human tumor profile dataset were ranked based on Pearson's correlation with the oncogene of interest (using only the primary PCA profiles and not profiles from metastatic or benign tissues). For genes in the experimental set of interest, the *t*-scores for the correlation coefficients in the ranked list were summed up; the significance of this sum was determined both by 1000 randomly selected gene sets (the Q1 hypothesis) and 1000 random permutations of the values for the reference gene (i.e. the oncogene used to rank the other genes) in the tumor dataset (the Q2 hypothesis). The sum of t-scores from the actual datasets were expressed as standard deviations from the mean of either the Q1 permutation results or the Q2 permutation results, with two-sided significance *p*-values determined assuming normal distributions of the permutation results. The higher of the Q1 and Q2 *p*-values determined enrichment (with both Q1 and Q2 required to move in the same direction).

The Entrez Gene identifier was used in mapping genes across the array datasets. For the prostate tumor datasets, where a gene was represented multiple times on a given platform, the “best” probe for the gene was selected in a manner not biased towards the direction of relative expression changes (the probe with the largest average signal intensity for the prostate tumor Affymetrix datasets, the probe with the least missing values across samples for the prostate cDNA microarray dataset). For the oncogenic signature gene sets, where a gene was represented by multiple array probes, the gene was represented at most once in a given set (it was possible within a signature for a given gene to be represented in both the “up” lists and the “down” lists by different probes, though this was the case for only a handful of genes). Where the probe selected to represent the reference oncogene or biomarker for ranking the entire gene population failed to show a significant association with the corresponding oncogenic signature, alternative probes representing the oncogene were tried.

## Supporting Information

Supplementary Data File S1 Lists of unique named genes in each of the oncogenic pathway signatures of Table 2
(2.53 MB XLS)

Supplementary Data File S2 Lists of the corresponding genes overlapping among the various oncogenic signatures.
(3.42 MB XLS)

Supplementary Data File S3 For each of the androgen, Myc, Src, HER2, EGFR, cyclin D1, and Akt pathways, lists of genes overlapping between the oncogenic signature and correlates of the corresponding oncogene or biomarker in human prostate tumors.
(1.41 MB XLS)
